# Impact of replacing powdered gloves with powder-free gloves on hand-hygiene compliance among healthcare workers of an intensive care unit: a quasi-experimental study

**DOI:** 10.1186/s13756-020-00877-5

**Published:** 2021-01-06

**Authors:** Mayra Gonçalves Menegueti, Fernando Bellissimo-Rodrigues, Marcia A. Ciol, Maria Auxiliadora-Martins, Anibal Basile-Filho, Silvia Rita Marin da Silva Canini, Elucir Gir, Ana Maria Laus

**Affiliations:** 1 Ribeirão Preto Nursing School, University of São Paulo, Campus Universitário, s/n Monte Alegre, Ribeirão Prêto, São Paulo 14048-900 Brazil; 2 Social Medicine Department, Ribeirão Preto Medical School, University of São Paulo, Ribeirão Prêto, Brazil; 3 Department of Rehabilitation Medicine, School of Medicine, University of Washington, Seattle, USA; 4 Intensive Care Division, Ribeirão Preto Medical School, University of São Paulo, Ribeirão Prêto, Brazil

**Keywords:** Hand hygiene, Powdered gloves, Powder-free gloves, Compliance, Alcohol-based hand-rub

## Abstract

**Background/objective:**

After wearing powdered gloves, healthcare workers (HCW) are supposed to wash their hands instead of using alcohol-based hand-rub (ABHR). Washing hands takes longer than using ABHR, and the use of powdered gloves may be an obstacle to hand-hygiene compliance. This study aimed to evaluate the impact of replacing powdered gloves with powder-free gloves on hand-hygiene compliance among HCW of an intensive care unit (ICU).

**Methods:**

A quasi-experimental study was conducted in a general ICU of a tertiary care university hospital in Brazil. From June 1st to July 15th, 2017, all HCW were provided with powdered latex gloves only for all clinical procedures. From July 15th to August 31st, 2017, HCW were provided with nitrile powder-free gloves only. Hand-hygiene compliance was assessed through direct observation, and evaluated according to the World Health Organization Hand Hygiene guidelines. We calculated that a sample size of 544 hand hygiene opportunities needed to be observed per period. Data analysis were performed using the STATA SE® version 14, and we compared the individual’s percentage of compliance using the t test for paired data before and after the intervention.

**Results:**

Overall, 40 HCW were assessed before and after the introduction of nitrile powder-free gloves, with 1114 and 1139 observations of hand hygiene opportunities, respectively. The proportion of compliance with hand hygiene was 55% (95% confidence interval [CI] 51–59%) using powdered latex gloves and 60% (95% CI 57–63%) using powder-free gloves. The difference in proportions between the two types of gloves was 5.1% (95% CI 2.5–7.6%, *p* < 0.001).

**Conclusion:**

Our data indicate that replacing powdered gloves with powder-free gloves positively influenced hand-hygiene compliance by HCW in an ICU setting.

## Background

Healthcare-associated infections (HAI) are a public health problem, and in developed countries, 5–19% of hospitalized patients are reported to contract a health care-associated infection [1].

In the United States (US), it was estimated that 4.5–5.7 million dollars are spent yearly for treatment of HAI, with an average of two million cases, resulting in 80 thousand deaths a year [2]. In 2015, in the US, 12,299 patients were surveyed in 199 hospitals. Of those, 394 patients presented at least one episode of HAI (3.2%; 95% Confidence Interval [95% CI] 2.9–3.5%) [3].

Silva (2005) [4] stated that 30% of all HAI can be prevented and that there is convincing evidence that hand hygiene is the most effective measure in preventing these events [5–8]. Alcohol-based hand-rub (ABHR) has been listed since 2002 as a standard solution for hand hygiene for health care workers (HCW), while hand washing is recommended when there is for visible dirty in the hand or the HCW comes in contact with patients with *Clostridium difficile* [9, 10].

ABHR is useful to improve compliance with hand hygiene since its use requires about one-third of the time to wash hands with soap and water. In addition, ABHR is more effective in eliminating microorganisms, and sometimes improves the cleanliness of the hands of HCW [10–16]. ABHR can be taken to the bedside, allowing workers to clean their hands while caring for patients. For the World Health Organization (WHO), hand-hygiene products must be easily accessible and placed as close as possible to the patient care area or where treatment is being delivered [10].

HCW have increasingly complied with hand hygiene practices when they are provided with ABHR and after capacity building training [17–19].

A study performed in an intensive care unit in the state of Virginia (United States), showed the benefit of ABHR for compliance with hand hygiene. After providing one ABHR for every four beds, hand hygiene rates went from 19 to 41% [20].

It is recommended that HCW wear gloves to prevent that microorganisms that are either colonizing or temporarily present in their hand skin be transmitted to patients or from one patient to another, while reducing the risk that HCW themselves acquire infections from the patients [21]. After removing the gloves, the HCW should perform hand hygiene. Since powdered gloves preclude the use of an ABHR after their removal, using powder-free gloves should be encouraged, because they do not interfere with hand-hygiene using ABHR [21].

The US Food and Drug Administration (FDA) prohibits the sales of powdered surgical gloves. In the past, these gloves were popular for being easier to put on and remove than powder-free gloves. However, recent studies have pointed out that the powders pose a substantial risk to develop allergies to them by the healthcare workers and patients [22].

Despite these data, in developing countries the use of powdered gloves is still a frequent practice, mainly due to the lower cost of these gloves. However, if the use of powdered gloves decreases compliance with hand hygiene, there might be an impact in HAI rates, possibly increasing them.

This study aimed to evaluate the impact of replacing powdered gloves with powder-free gloves on the hand-hygiene compliance among HCW of an intensive care unit (ICU) in Brazil.

## Methods

### Type of study

The study was quasi-experimental and quantitative, carried out in a nine-bed intensive care unit, in a tertiary teaching hospital. This study was approved by the research ethics committee of the institution under the number CAAE 69241417.8.0000.5440. All HCW signed an informed consent before participating in the study.

The units of analysis were the opportunities for hand hygiene of all professionals working in the participating ICU.

The sample size was estimated using the STATA SE® version 14. We considered that an overall increase in compliance of 10% from the pre-intervention phase (powdered gloves) to the intervention phase (powder-free gloves), would be worth to be detected. Sample size for comparison of two percentages are larger when the percentages approach 50%. We calculated the largest sample size needed to detect a 10% change in that situation (in our case, corresponding to 45% compliance in the pre-intervention and 55% in the post-intervention), with a significance level of 0.05 and power of 0.90. Under those assumptions, we would need 544 opportunities per period: pre-intervention (1 month) and intervention (1 month), totaling 1088 overall opportunities.

### Data collection procedure

A checklist was used to assess hand hygiene practices of all observed employees. The main author, who was personally trained by the infection control team of the Hôpitaux Universitaires de Genève, was responsible for observing the compliance with hand hygiene, without interfering in the HCW routine. Hand hygiene opportunities were classified according to the WHO five moments concept [10]: before contact with patients, before the aseptic procedure, after body fluid exposure, after contact with patients, and after contact with patients' environments. For each opportunity, compliance (yes or no) was marked in the checklist.

The study comprised two phases. In phase I, all HCW working in the ICU wore powdered gloves, when necessary, and were observed for hand hygiene practices in all shifts. In phase II, all HCW in the ICU wore powder-free gloves, when necessary, and hand hygiene practices were observed in all shifts. Each HCW was observed for at least 17 opportunities (maximum of 41) for hand hygiene during phase I, and for at least 19 (maximum of 37) during phase II. At the end of the study, all participants were asked which kind of gloves they preferred to use, and why.

### Statistical analysis

All data collected were entered into an Excel spreadsheet and later analyzed using the STATA SE® version 14. Descriptive statistics were calculated to describe the characteristics of the 40 HCW of the ICU.

The data was analyzed by individual health care professional and by hygiene opportunity. Overall, the percentage of compliance was the number of times in which hand hygiene was performed divided by the total number of hygiene opportunities within the period of time a certain type of glove was used, and multiplied by 100%. Similarly, in the individual level, compliance was calculated as number of times a HCW performed hand hygiene divided by the number of opportunities the individual had to do so, multiplied by 100%. We compared the overall compliance between the two periods using a test of proportion. We compared the individual’s percentage of compliance using the t test for paired data. We also calculated the 95% confidence interval (CI) for the proportion of compliance among the individuals. Results were considered statistically significant at 0.05 level. The answers regarding gloves preferences were tabulated and presented in frequency of each option.

## Results

Forty HCW were observed during the study. Additional file 1: Table S1 describes their demographic characteristics.

Among the 40 assessed HCW, a total of 1114 and 1139 hand hygiene events were observed for powdered and powder-free gloves, respectively. Additional file 1: Table S2 shows the hand-hygiene compliance percentage for each opportunity category and in each studied phase, considering the opportunity as the unit of analysis. We ordered the types of opportunity by decreasing percentage of total compliance. After contact with fluids, compliance percentages remained the same and ABHR use increased slightly; however, ABHR use was low at this hand hygiene type of opportunity, while compliance percentage were almost 100%. After contact with patients, general hand-hygiene compliance percentage as well as use of ABHR with powdered gloves was higher than with powder-free gloves. Before aseptic procedures, there was an increase in general hand-hygiene compliance percentage, and ABHR use was four times higher when using powder-free gloves. After contact with surfaces, there was an increase in compliance percentage and ABHR use when using powder-free gloves. Before contact with patients, the percentage of compliance with hand hygiene (hand wash or ABHR) was low with powdered gloves (18.5%) and increased with the use of powder-free gloves (31.2%), although this is still considered a low percentage in a hospital setting. ABHR use was 2.3 times higher for the powder-free gloves. Finally, overall, there was an increase in compliance using ABHR when wearing the powder-free gloves, while compliance by washing hands remained somewhat stable, except for before aseptic procedures, when there was more compliance with hand washing for powdered gloves.

**Fig. 1:**
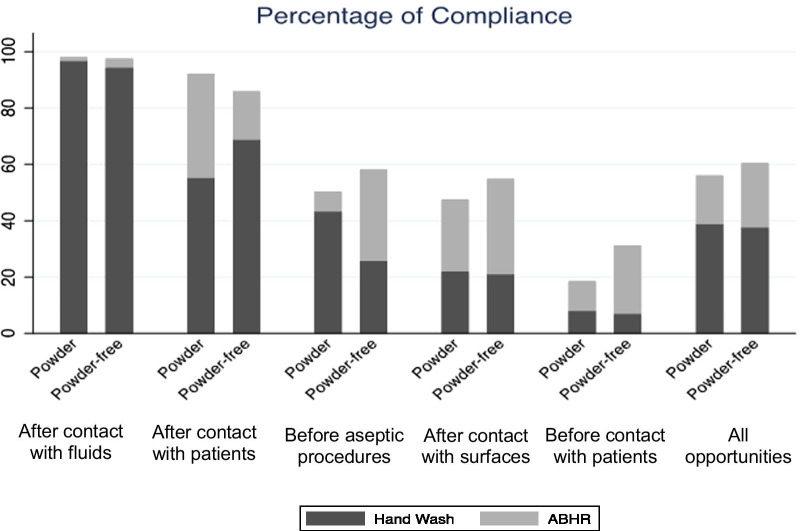
Percentages of compliance with hand hygiene practices while in used of powdered or powder-free gloves, overall and by type of opportunity

Figure 1 displays the overall percentages of compliance with hand hygiene practices while wearing powdered and powder-free gloves, ordered by decreasing percentage of compliance according to opportunity and all opportunities together.

When treating the individual as the unit of analysis, we looked only at the total compliance of the individual with powdered and powdered-free gloves, since each person had a small number of events in each opportunity category and those percentages would not be robust estimates of the true proportion of compliance.

**Fig. 2:**
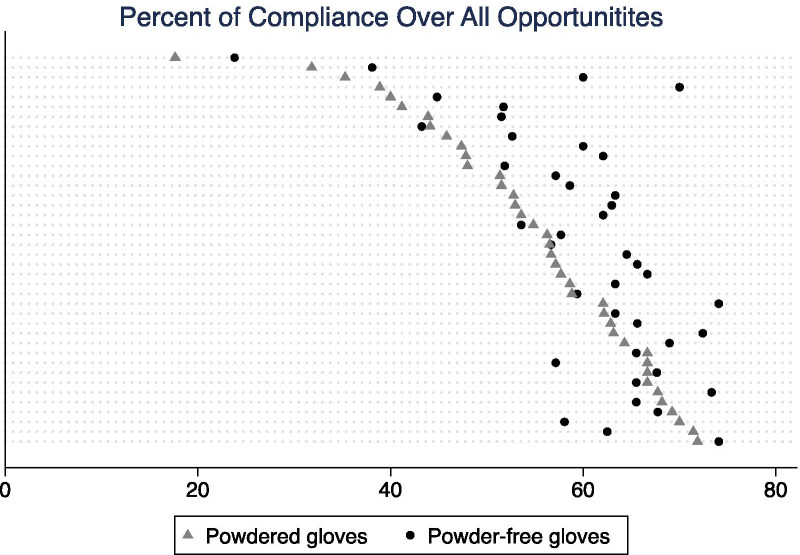
Compliance with hand hygiene for each studied healthcare workers, wearing each type of gloves

Figure 2 shows the total percentage of hand-hygiene compliance for each HCW, wearing powdered and powder-free gloves. Most individuals had a higher rate of compliance with hand hygiene when they were provided with the powder-free gloves. The mean percentage of compliance with hand-hygiene was 55.0% (95% CI 51.1–58.9%) with powdered gloves and 60.1% (95% CI 56.9–63.3%) with powder-free gloves. The mean difference in percentages between hand-hygiene compliance for both types of gloves for the individuals was 5.1% (95% CI 2.5–7.6%), and it was statistically different from 0 (*p* < 0.001), with higher proportion of compliance when using the powder-free gloves.

In our study, forty HCW (100%) reported preference for powder-free gloves because they cause less dry skin when compared to powdered ones. They also reported regular nasal discomfort 25 (62.5%) due to powder inhaling when wearing powdered gloves. When comparing vinyl and latex gloves, forty HCW (100%) reported no preference since both are resistant to care performance.

## Discussion

The literature has clearly shown the relevance of hand hygiene as the main measure for preventing HAI. Although it the practice of hand hygiene is well stablished and disseminated, ensuring its compliance in different healthcare situations is still a challenge. Therefore, strategies to increase such compliance are relevant to hospitals.

In this study, we observed an overall percentage of compliance of 55.9% using the powdered gloves and 60.4% using the powder-free gloves. Our observations are similar to other studies, although they did not reported whether gloves were powdered or not. In a study at the emergency department of a university hospital in Brazil, a hand-hygiene percentage of compliance of 54.2% was observed among 59 healthcare workers [23]. In another quasi-experimental study carried out in 55 departments of 43 hospitals in Costa Rica, Italy, Mali, Pakistan, and Saudi Arabia, a total of 21,884 hand-hygiene opportunities were identified, with compliance of 51% of the time [24].

A study performed in five ICU of four hospitals in Texas evaluated a total of 3620 opportunities for hand hygiene, which were recorded during 18 days of observation (144 h), and a proportion of compliance of 64% was observed [25].

After implementing the use of powder-free gloves in the ICU, we observed an increased hand-hygiene percentage of compliance of 60%, which was statistically significant, but still modest. The possibility of using ABHR after removing the powder-free gloves may have contributed to such an increase in the percentage. During health care, it is often unfeasible for clinicians to sanitize hands with soap and water. For instance, while bathing a patient, HCW perform intimate hygiene and then continue bathing the patient; in this case, they would need to remove gloves and apron to wash their hands. If they were wearing powder-free gloves, they could have removed them and cleansed their hands with ABHR from the bedside, what would encourage the practice of hand hygiene.

We observed an increase in ABHR use before contact with patients, thus increasing compliance in this opportunity. This fact can be explained considering that a professional when examining the patient can easily remove powder-free gloves, use the ABHR and examine another patient. With powdered gloves, he would need to wash his hands with soap and water, which would take more time and considering the countless opportunities for hand hygiene that occur in the ICU, it could not be feasible.

We also observed an increase in ABHR use before aseptic procedures. In the study ICU, many patients are under contact precautions and, normally, HCW come into a general contact with them using gloves and, after that, should change gloves and perform hand hygiene before an aseptic task. That indication is truly facilitated by the application of ABHR rather than the traditional handwashing, which could not happen if the HCW was using powdered gloves.

A study carried out in Brazil showed a hand-hygiene percentage of compliance of 18.4% before aseptic procedures. The authors point out that the main reason might be that healthcare workers wears gloves and assumes that he does not need to perform hand hygiene [26]. It is possible that some HCW believe that wearing gloves can dispense with the need for hand hygiene. However, it is also true that there may be a difficulty in performing hand hygiene when using powdered gloves.

After contact with fluids and/or secretions, no significant changes were noted for hand-hygiene compliance or for ABHR use. However, hand hygiene was high (almost 100%) for either type of glove, showing that HCW are indeed concerned with their exposure to diseases after completion of procedures that included contact with fluids [27]. In a study in a neonatal ICU in Brazil, HCW avoided ABHR after performing procedures, using ABHR only 1.7% of opportunities, while general compliance hand hygiene was 61.7% [28].

After contact with patients, hand-hygiene compliance was slightly lower with powder-free than with powdered gloves. The proportion of use of ABHR was also reduced.

Hand hygiene after contact with surfaces increased slightly, as well as ABHR use when using powder-free gloves. When touching surfaces, HCW may have a false sense of reduced risk of contamination, since they are not touching the patient or body fluids directly, leading them to assume that they could use ABHR instead of hand washing. The use of powder-free gloves enables the provider to make that choice.

Our data suggests that the use of ABHR is facilitated when HCW use powder-free gloves rather than powdered gloves. Controversially, however, the frequency of using ABHR in the moments nº 3 and 4 fell with the changing from powdered to powder-free gloves. We cannot assure what is the real reason for that but we believe this is a cultural thing. HCW apparently feel more comfortable washing hands after touching the patient or their body fluids. Others have reported similar findings [29, 30].

We also observed that HCW preferred using powder-free gloves, mainly due to a less intense drying effect on the hands skin than the observed with the use of powdered gloves. In pandemic times, such as nowadays, HCW have been compelled to use gloves more frequently and for longer periods, leading to an increase in the frequency of related adverse events [31]. Therefore, the use of powder-free gloves may be beneficial in this regard too.

One of the limitations of this study was that we only tested the gloves in one ICU unit with 40 HCW. It would also be important to have a longer period of observation (more event opportunities) as well as some measures of comfort and hand conditions that could evaluate not only the compliance with hand hygiene, but also the effects on the HCW hand skin. Another potential limitation is the possibility of time-dependent bias, inherent in quasi-experimental trials. However, the *Hawthorne effect* regarding hand hygiene observation tends to vanish with time [32, 33]. Therefore, we would expect, in this case, that compliance would spontaneously only decrease over the study phases, and not increase, as observed. That is why we have chosen the study sequence: powdered gloves followed by powder-free gloves and not the opposite.

## Conclusion

Our data indicate that replacing powdered gloves with powder-free gloves had a positive influence on hand-hygiene compliance among the HCW in the studied intensive care unit.

## Supplementary information

**Additional file 1**. **Table S1**: Demographic aspects of the healthcare workers participating in the study. **Table S2**: Hand-hygiene compliance percentages for each opportunity category with powdered latex and powder-free nitrile gloves.

## Data Availability

All data generated or analyzed during this study are included in this published article. The anonymized datasets analyzed during the current study are available from the corresponding author (MGM; mayramengueti@eerp.usp.br) on reasonable request, as long as this meets local ethics and research governance criteria.

## Abbreviations

HCW: Healthcare workers
ABHR: Alcohol-based hand-rub
ICU: Intensive care unit
WHO: World Health Organization
HAI: Healthcare-associated infections
95% CI: 95% Confidence interval
FDA: Food and Drug Administration
N: Number
