# Supplementary material for: Impact of replacing powdered gloves with powder-free gloves on hand-hygiene compliance among healthcare workers of an intensive care unit: a quasi-experimental study
Source: Antimicrob Resist Infect Control. 2021 Jan 6;10:6. doi: 10.1186/s13756-020-00877-5 (PMC7789181; doi:10.1186/s13756-020-00877-5)
Supplement: Supplementary file 1 — Additional file 1. Table S1: Demographic aspects of the healthcare workers participating in the study. Table S2: Hand-hygiene compliance percentages for each opportunity category with powdered latex and powder-free nitrile gloves. [file 13756_2020_877_MOESM1_ESM.docx]

Table S1. Demographic aspects of the healthcare workers participating in the study

| **Demographic feature (n = 40)** |  |
| --- | --- |
| **Age**, years, mean (standard deviation) | 39.0 (9.2) |
| **Sex, female,** n (%) | 28 (70.0) |
| **Profession,** n (%) |  |
| Auxiliary nurse | 28 (70.0) |
| Registered Nurse | 5 (12.5) |
| Medical Doctor | 4 (10.0) |
| Physiotherapist | 3 (7.5) |
| **Working time,** n (%) |  |
| Full-time | 18 (45.0) |
| Part-time (50-90%) | 22 (55.0) |
| **Working shift** |  |
| Day shift | 26 (65.0) |
| Night shift | 14 (35.0) |

Table S2. Hand-hygiene compliance percentages for each opportunity category with powdered latex and powder-free nitrile gloves.

| Opportunity category |  | Powdered latex gloves | Powder-free nitrile gloves |
| --- | --- | --- | --- |
| Before contact with patients  (Moment 1) | Number of opportunities | 352 | 359 |
|  | Use of Alcohol-based hand-rub, n (%) | 37 (10.5) | 87 (24.2) |
|  | Use of water and antibacterial, n (%) | 28 (8.0) | 25 (7.0) |
|  | Total compliance, n (%) | 65 (18.5) | 112 (31.2) |
| Before aseptic procedures (Moment 2) | Number of opportunities | 177 | 198 |
|  | Use of Alcohol-based hand-rub, n (%) | 12 (7.0) | 64 (32.3) |
|  | Use of water and antibacterial, n (%) | 77 (43.3) | 51 (25.8) |
|  | Total compliance, n (%) | 92 (50.3) | 115 (58.1) |
| After contact with fluids (Moment 3) | Number of opportunities | 153 | 162 |
|  | Use of Alcohol-based hand-rub, n (%) | 2 (1.3) | 5 (3.1) |
|  | Use of water and antibacterial, n (%) | 148 (96.7) | 153 (94.4) |
|  | Total compliance, n (%) | 150 (98.0) | 158 (97.5) |
| After contact with patients (Moment 4) | Number of opportunities | 255 | 234 |
|  | Use of Alcohol-based hand-rub, n (%) | 94 (36.8) | 40 (17.1) |
|  | Use of water and antibacterial, n (%) | 141 (55.3) | 161 (68.8) |
|  | Total compliance, n (%) | 235 (92.1) | 201 (85.9) |
| After contact with surfaces (Moment 5) | Number of opportunities | 177 | 186 |
|  | Use of Alcohol-based hand-rub, n (%) | 45 (25.5) | 63 (33.8) |
|  | Use of water and antibacterial, n (%) | 39 (22.0) | 39 (21.0) |
|  | Total compliance, n (%) | 84 (47.5) | 102 (54.8) |
| All opportunities | Number of opportunities | 1114 | 1139 |
|  | Use of Alcohol-based hand-rub, n (%) | 190 (17.1) | 259 (22.7) |
|  | Use of water and antibacterial, n (%) | 433 (38.9) | 429 (37.7) |
|  | Total compliance, n (%) | 623 (55.9) | 688 (60.4) |
